# Enhanced Anti-Inflammatory Effect of the Combination of *Lactiplantibacillus plantarum* LS/07 with Methotrexate Compared to Their Monotherapies Studied in Experimental Arthritis

**DOI:** 10.3390/molecules28010297

**Published:** 2022-12-30

**Authors:** Katarína Pružinská, Lukáš Slovák, František Dráfi, Silvester Poništ, Ivo Juránek, Martin Chrastina, Karol Švík, Ladislav Strojný, Ľuboš Ambro, Katarína Bauerová

**Affiliations:** 1 Centre of Experimental Medicine, Institute of Experimental Pharmacology and Toxicology, Slovak Academy of Sciences, Dúbravská cesta 9, 84104 Bratislava, Slovakia; 2 Jessenius Faculty of Medicine in Martin, Comenius University, Malá Hora 4A, 03601 Martin, Slovakia; 3 Center of Clinical and Preclinical Research, Faculty of Medicine, Pavol Jozef Šafárik University, Trieda SNP 1, 04011 Košice, Slovakia; 4 Center for Interdisciplinary Biosciences, Technology and Innovation Park, Pavol Jozef Šafárik University, Jesenná 5, 04001 Košice, Slovakia

**Keywords:** adjuvant arthritis, probiotics, *Lactobacilli*, *Lactiplantibacillus plantarum* LS/07, methotrexate, inflammation, immune modulation

## Abstract

The gut microbiome (GM) of rheumatic arthritis (RA) patients is often altered in composition and function. Moreover, methotrexate (MTX), one of the most frequently used disease-modifying antirheumatic drugs, is known to negatively affect GM composition. The modulation of immune system activity is one of the therapeutic benefits of probiotics. The aim of the current investigation was to determine the impact of MTX therapy combined with one of the Lactobacillus strains, *Lactoplantibacillus plantarum* LS/07 (LB), on adjuvant arthritis (AA) in rats. Methods focused on biometric and inflammatory parameters in AA, particularly on plasmatic levels of IL-17A, MMP-9, and MCP-1, and the activities of gamma-glutamyl transferase in the spleen and joints were applied. Enhancing the effect of MTX, LB positively influenced all biometric and inflammatory parameters. The findings of the present study may be of help in proposing novel therapeutic strategies for RA patients.

## 1. Introduction

Production of autoantibodies is involved in chronic joint inflammation in rheumatoid arthritis (RA), a systemic autoimmune disease. The lungs, heart, and kidneys have been reported to be also affected by RA [1]. Interaction between the human leukocyte antigen gene (HLA) and environmental factors is one of the triggers leading to RA. Smoking, illness, and recently reported dysbiosis belong to the environmental factors [2,3,4].

Probiotics are live bacteria that, given to host in appropriate doses, provide significant health benefits [5]. As nutrition is an important environmental factor influencing the gut microbiota (GM) function, current research focuses on its involvement in the etiology, development, and activity of many diseases, including rheumatic illnesses [6]. Exogenous bacteria may exert subject-specific impacts on GM via altering dysbiosis-related conditions such as RA, which may be improved [7]. Probiotic bacteria have been suggested as an effective tool to treat gut dysbiosis and/or better the homeostasis of GM; hence, having a potential of positively influencing systemic immune responses, probiotics have been proposed for the use as adjuvant treatment in treating the immune system-related disorders [1,8].

Probiotics have been studied to identify potential beneficial effects in the prevention and treatment of various systemic conditions in both animal studies and human trials. The conditions comprise inflammatory and autoimmune diseases such as RA, multiple sclerosis, ulcerative colitis, and hepatic encephalopathy. Positive modulation of immune system activity by specific bacteria strains is of the main benefits of probiotics. Various probiotic strains are being used, and various effects are observed [9]; hence, a suitable strain needs to be chosen each time [10]. Some strains have been shown to stimulate the immune system and enhance immune response, which is desirable for immunodeficient patients [11]. For people with immuno-activating diseases such as RA, other strains have been reported to be beneficial due to inhibiting the immune response [2,12].

Although the benefits of probiotics were typically both disease- and strain-specific, meta-analyses of studies using several strains indicated that some effects are shared by various strains [13]. Probiotics are believed to have various health effects, including the ability to modulate the immune response, support gut barrier integrity, and modulate GM. Probiotics may influence existing bacterial communities either directly through trophic interactions or indirectly by changing the production of molecules derived from the host, or both [14,15,16]. Current studies suggested that drugs and genetic material have a reciprocal relationship in which they can affect one another and impact therapeutic results [17].

The most commonly used strains, whether in a mixed culture or as a single species, are *Lactobacilli*, *Bifidobacteria*, and *Streptococci*. A number of randomized controlled clinical studies and meta-analyses on infectious illnesses, antibiotic-associated diarrhea, irritable bowel syndrome, abdominal pain, and colitis have recognized and supported their beneficial effects [18,19].

The GM of RA patients has been found to be altered in composition and function [20], with a substantial reduction in microbial diversity compared to healthy controls [21]. Increased disease duration in RA patients was associated with decreased GM diversity [21]. *Faecalibacterium* is one of the most prevalent butyrate-producing *Firmicutes* in the human gut. RA patients have been found to have lower levels of *Faecalibacterium* and other butyrate-producing taxa such as *Flavobacterium* [22]. Compared to healthy controls, the GM of RA patients exhibited a considerable increase in the order of *Lactobacillales* [21,22] and a broader range of *Lactobacilli* [23]. Intriguingly, administration of some *Lactobacillus* species has been reported to improve RA clinical symptoms, indicating that distinct *Lactobacilli* may play diverse roles in RA etiology and disease activity control [24,25].

There is a certain inconsistency in studies reporting different effects of probiotics on the activity of RA [26]. Zamani et al. (2016) have reported that probiotic supplementation improved the disease activity scores (looking at 28 joints) in patients suffering from RA compared to placebo group [27]. Chen et al. (2016) examined the gut microbiota profile of 40 RA patients and 32 healthy controls. They observed a lesser diversity of gut microbes in RA patients compared to controls and revealed a close correlation between the severity of the disease and the quantity of rheumatoid factor in serum [21].

An interaction between the GM and local/systemic immunity, as well as activation of joint inflammation, has been pointed out in animal studies [28]. One of the many studies in mice sensitive to collagen-induced arthritis (CIA) has documented an increase in the *Lactobacillaceae* family and the *Lactobacillus* genus [29]. In another type of inflammation animal model, in DSS-induced mouse models of ulcerative colitis, *L. acidophilus* treatment suppressed cell-mediated secretion of proinflammatory cytokines/interleukins (IL-1β, IL-6, TNF-α, IL-17, and IL-23) activated by Th17 and supported secretion of the anti-inflammatory cytokine IL-10 [30].

*Lactiplantibacillus plantarum* LS/07 (LB) was studied on animal models and published articles state its potential to exert a preventive effect on colon carcinogenesis [31,32,33,34], mammary carcinogenesis, and immunomodulation [35,36], influencing lipid profiles in rats receiving high-fat diet by improving the lipid profile and therefore contributing to a healthier bowel microbial balance [37]. Other animal studies showed the improvement of intestinal inflammation in rats via the increased production of anti-inflammatory cytokines [38,39,40,41]. LB demonstrated the best results in *in vitro* testing against pathogenic enterotoxigenic *E. Coli*; compared to other strains of *lactobacilli*, it showed the best inhibitory effect against the multiplication (growth) of this pathogenic strain [34].

Methotrexate (MTX), one of the most used disease-modifying anti-rheumatic drugs (DMARDs), has been reported to alter GM composition, partially restoring the microbial balance disrupted due to illness [20,22]. Profound knowledge in GM modifications in patients undertaking complex therapy regimens might help better their outcomes via customized therapeutic approaches [22]. In fact, GM modulation has been indicated for rheumatic disease prevention and control [42].

Considering the occurrence of disrupted GM in the etiology of RA, clinical interest in probiotics to address gut dysbiosis and downregulate the proinflammatory cytokine cascade implicated in inflammatory arthritis has grown [12,43,44]. *L. casei* has been shown to help reduce RA symptoms and inhibit proinflammatory cytokines in patients taking DMARDs, likely implying a favorable synergistic impact of DMARDs and probiotics [24,45].

Based on the positive influence of the *Lactobacillacea* family on microbiota in RA patients, we have decided to study in detail the effect of one of the *Lactobacillus* strains, *L. plantarum* LS/07, in combination therapy with MTX in adjuvant arthritis in rats. We provide experimental evidence that is supportive for the use of LB in complex therapy of RA, particularly in combination with MTX.

## 2. Results

### 2.1. Biometric Parameters in Adjuvant Arthritis

**Figure 1:**
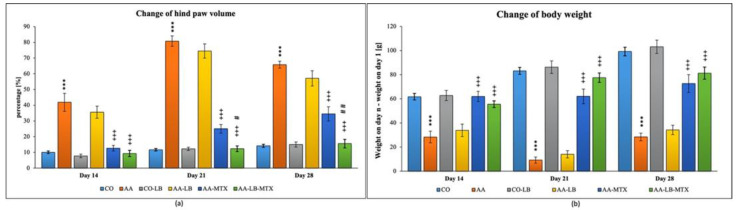
Change of hind paw volume (cHPV) (**a**) and change of body weight (cBW) (**b**) were assessed on experimental days 14, 21, and 28 in rats. CO—healthy control group, AA—group of controls with adjuvant arthritis (AA), CO-LB—a control group of healthy animals administered with *L. plantarum* (LB), AA-LB—group of animals with AA administered with LB, AA-MTX—rats with AA treated with methotrexate (MTX), AA-LB-MTX—group of rats with AA receiving combinational therapy of MTX and LB. Values are expressed as mean ± S.E.M. Statistical significance was evaluated by applying ANOVA for independent variables: *** *p* < 0.001 vs. CO; +++ *p* < 0.001 vs. AA; # *p* < 0.05 and ## *p* < 0.01 vs. AA-MTX.

In our experiment with Lewis rats, we chose two biometric parameters for describing the development of experimental arthritis and further for evaluation of the treatment effectiveness. Figure 1 presents the obtained data for the change of hind paw volume (cHPV) (Figure 1a) and the change of body weight (cBW) (Figure 1b). The arthritis was already developed on day 14. For both parameters, we obtained significant differences between arthritis and healthy animals. For cHPV we observed an increase in this parameter for animals suffering from arthritis compared to the healthy control group (Figure 1a). In contrast, for cBW we observed a decrease (Figure 1b). After 1 week, in the case of both parameters, were the differences between healthy and arthritic animals even more profound, which is in accordance with the acute phase of the arthritis development in AA. During the chronic phase of arthritis, which is apparent on experimental day 28, we observed regression of the differences between AA and healthy control animals. The administration of LB to healthy control did not show any effect on monitored biometric parameters. LB administered to arthritic animals slightly improved both parameters, but it had a more visible effect for cHPV than cBW during the whole experiment (Figure 1a,b). The administration of MTX in monotherapy caused significant alleviation of biometric parameters in arthritic animals compared to untreated animals. LB administered in combination with MTX caused improvement in the therapy efficacy for both biometric parameters and all experimental days (Figure 1a,b). Moreover, we measured significant change for cHPV on experimental days 21 and 28 (Figure 1a).

### 2.2. Activity of Gamma-Glutamyl Transferase in Spleen and Joint

**Figure 2:**
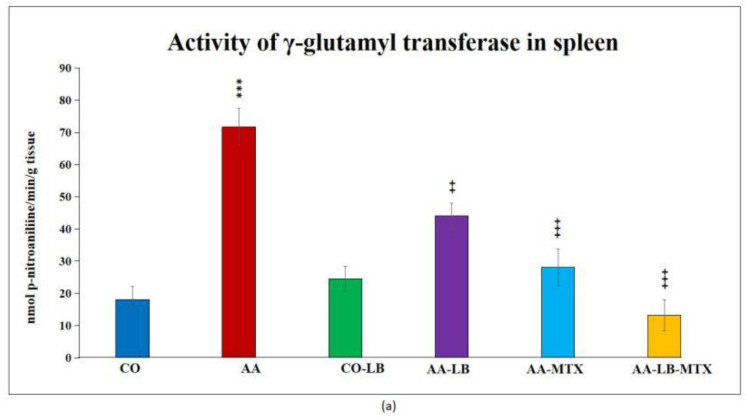

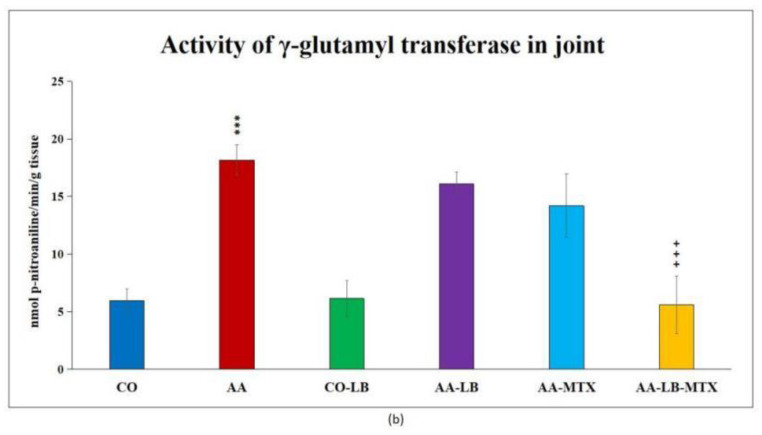
The effect of *L. plantarum* administered alone or in combination with MTX on the activity of GGT in the spleen (**a**) and joint (**b**) on day 28. CO—healthy control group, AA—group of controls with adjuvant arthritis (AA), CO-LB—a control group of healthy animals administered with *L. plantarum* (LB), AA-LB—group of animals with AA administered with LB, AA-MTX—rats with AA treated with methotrexate (MTX), AA-LB-MTX—group of rats with AA receiving combinational therapy of MTX and LB. Values are expressed as mean ± S.E.M. Statistical significance was evaluated by applying ANOVA for independent variables: *** *p* < 0.001 vs. CO; +++ *p* < 0.001 and ++ *p* <0.01 vs. AA.

At the end of the experiment, we collected relevant tissues to monitor gamma-glutamyl transferase (GGT) activity. Although the values were elevated differently in the studied tissues (higher values were measured in the spleen compared to joint tissue), significant differences were shown between arthritic and healthy animals (Figure 2a,b) in both analyzed tissues. Again, as for biometric parameters as for the activity of GGT, it did not prove any change in control groups administered with LB compared to untreated healthy controls. In both tissues, we could observe the same pattern in decreasing the GGT activity after administration of LB to arthritic animals in monotherapy as well in combination with MTX: the highest activity was measured in AA-LB group, following AA-MTX. The combination therapy (AA-LB-MTX) decreased the activity of GGT most effectively and significantly.

### 2.3. Immunological Parameters in Adjuvant Arthritis and Their Modulation by L. plantarum and Its Combination with Methotrexate

**Figure 3:**
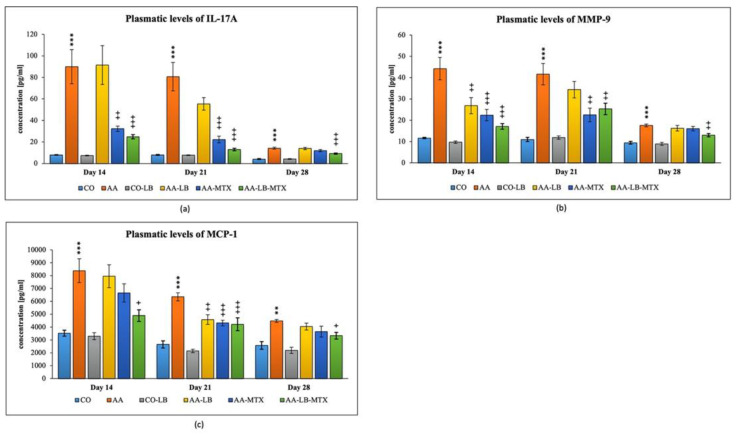
The effect of *L. plantarum* on several biochemical markers such as IL-17A (**a**), MMP-9 (**b**), and MCP-1 (**c**), was measured on different experimental days 14, 21, and 28. CO—healthy control group, AA—group of controls with adjuvant arthritis (AA), CO-LB—a control group of healthy animals administered with *L. plantarum* (LB), AA-LB—group of animals with AA administered with LB, AA-MTX—rats with AA treated with methotrexate (MTX), AA-LB-MTX—group of rats with AA receiving combinational therapy of MTX and LB. Values are expressed as mean ± S.E.M. Statistical significance was evaluated by applying ANOVA for independent variables: *** *p* < 0.001, ** *p* < 0.01 vs. CO group; +++ *p* < 0.001, ++ *p* <0.01, + *p* < 0.05 vs. AA group.

We selected three different inflammatory markers to evaluate the intensity of inflammation and its modulation by the studied therapy. In arthritic animals, the concentration profile for metalloproteinase-9 (MMP-9) and monocyte chemotactic protein-1 (MCP-1) in plasma follows a decreasing trend from day 14 to day 28 (Figure 3b,c). On the other hand, for interleukin-17A (IL-17A), we observed a different pattern with the peak on day 21 and minor differences apparent on day 28 (Figure 3a). All parameters were not changed if we compared the healthy untreated group and the healthy group treated with LB (Figure 3a–c). In the case of IL-17A, the administration of LB improved the elevated concentration in arthritic animals only on day 21 during the acute phase of AA, however the significance was not proved. MTX was effective in lowering the IL-17A concentrations for the first two experimental weeks but not on day 28. The combination with LB significantly lowered IL-17A levels during the whole experiment (Figure 3a). For arthritis, increased concentrations of MMP-9 and MCP-1 in plasma were affected therapeutically mainly at day 14. The increasing efficacy was in this order: AA-LB followed by AA-MTX and AA-LB-MTX. The addition of LB to MTX caused therapeutic improvement, although not significantly between AA-MTX and AA-LB-MTX (Figure 3b,c). MMP-9 was further improved on day 21 by MTX and its combination with LB. However, on day 28, only the combination therapy was effective (Figure 3b). MCP-1 on day 21 was decreased comparable in all treated groups, with significance for AA-MTX and AA-MTX-LB. A slight difference between the healthy control and arthritic group on day 28 was found. Similarly, there were no differences among treated groups of arthritic animals (Figure 3c).

## 3. Discussion

Probiotics have been recently studied mainly for their modulating effects on the immune system and inflammation. The mechanisms of their action involve downregulation of toll-like receptors (TLRs); alteration of the production of cytokines by antigen-presenting cells (APCs), which trigger adaptive responses; enhancing the differentiation of B cells into plasma cells; competing with pathogens in the intestinal mucosa by entering the lamina propria; and adhering to epithelial cells causing initiation of signaling cascade that leads to immune regulation [46,47,48,49,50,51,52,53,54,55]. Probiotic bacteria stimulate cells responsible for innate and acquired immunity, such as epithelial cells and dendritic cells (DCs), natural killer cells (NK), macrophages, and lymphocytes, effectively modifying immune system activity [46,47,48]. Primary response to pathogens mediated by the innate immune system is generated after activation of pattern recognition receptors (PRRs) that are expressed on immune and non-immune cells, including NK cells, DCs, macrophages, fibroblasts, and epithelial cells [46,48,49]. Most studied PRRs are toll-like receptors (TLRs) responsible for activation of signaling pathways regulating cell proliferation and cytokine production [48,50]. It has been demonstrated that probiotics via downregulation of TLR expression can reduce inflammation [51]. Adaptive immune responses rely mostly on T cells [49]. T-helper as Th1 and Th17 cells mediate inflammatory responses that protect the host from the infection; their overactivation may yet result in harmful inflammation [10]. Probiotics have been shown to modulate production of cytokines by antigen-presenting cells (APCs), triggering adaptive responses [52]. Beyond the immunomodulatory capabilities of DCs and T cells, specific probiotic strains enhance the differentiation of B cells into plasma cells resulting in increased secretion of IgA [53]. Secretory IgA protects against infections by blocking bacterial adhesion to the epithelium hence preventing host tissue penetration [52,53]. Competitive exclusion, where probiotics cling to the intestinal mucosa and prevent pathogens from entering the lamina propria, is an essential mechanism by which probiotics compete in the host environment [53,54]. Additionally, probiotic microbe adherence to epithelial cells may initiate a signaling cascade leading to improved immune regulation [55].

The gut epithelium, immune system, and commensal bacteria interact together in modulating systemic inflammatory status [53]. RA pathogenesis is characterized by an imbalance between anti-inflammatory and proinflammatory cytokines (interleukin (IL)-1β, tumor necrosis factor-α (TNF-α), interferon (IFN)-y, IL-6, IL-12, and IL-17), all playing a significant role in the inflammatory processes involved in the pathology [56,57,58].

The proposed gut-joint axis mechanism of inflammatory arthritis is linked to the gut wall’s hyperpermeability, which can expose the immune system to microorganisms, triggering a systemic immune response that initiates a local inflammatory process within the joints [59,60]. In their clinical study, Alipour et al. (2014) showed that *L. casei* 01 supplementations decreased serum high-sensitivity C-reactive protein (CRP) levels, reduced tender and swollen joint counts, and improved global health scores (p < 0.05), and compared to controls, levels of interleukins (IL-10, IL-12) and TNF-α in the circulation were improved in the probiotic group [24]. Based on meta-analysis of randomized clinical studies covering effects of *Lactobacillus* as a single species or in mixed cultures with *Bifidobacterium* species, the probiotic supplementation lowered serum levels of IL-6 [12]. Another systematic review and meta-analysis evaluating the effectiveness of *L. casei* supplementation in RA revealed that this specific strain significantly reduced CRP levels [61].

Numerous animal studies have pointed out an interaction between the GM and local/systemic immunity as well as the activation of joint inflammation [28]. Some studies focusing on *Lactobacilli* effects were performed using collagen-induced arthritis (CIA) in rats or mice. In mice sensitive to CIA, an increase in the *Lactobacillaceae* family and the *Lactobacillus* genus has been reported [29]. Therapy with *L. casei* or *L. acidophilus* in a preclinical model of CIA in rats for 28 days was as effective as treatment with the standard antiarthritic drug indomethacin in terms of downregulation of proinflammatory cytokines and upregulation of anti-inflammatory cytokines in serum. The administration of *L. casei* and *L. acidophilus* significantly decreased parameters of oxidative stress in synovial effusate and ameliorated the arthritis score, hence pointing out the *Lactobacillus’s* anti-inflammatory and antioxidant properties [62]. The anti-inflammatory effects of *L. casei* in the CIA rat model were demonstrated by inhibiting COX-2 enzyme and downregulating proinflammatory cytokines [58].

In CIA in female Wistar rats, several *Lactobacillus* spp. including *L. plantarum* were examined. Mainly *L. casei*, *L. reuteri*, *L. fermentum* and *L. rhamnosus* helped to attenuate arthritis in this model by inhibiting proinflammatory cytokines and anti-CII-antibodies and through rebalancing of GM and metabolites such as short-chain fatty acids. *L. reuteri* and *L. casei* slowed down the Th1 immune response, while *L. rhamnosus* and *L. fermentum* impaired Th17 responses. However, *L. plantarum* did not alleviate arthritis even though it suppressed Th1 and Th17 immune responses, while *L. salivarius* only prolonged the onset of arthritis without influencing the immune response. *Lactobacilli* protect against CIA through both common and individual pathways [63].

Evaluation of *Lactobacilli* in adjuvant arthritis (AA) is rarely documented. Pan et al. (2019) used AA for the evaluation of *L. casei* effect in comparison to MTX. Administration of *L. casei* at the beginning phase of AA in Sprague-Dawley rats slowed down the development of arthritis in a way that was comparable to MTX, with normalization of GM, providing relief of arthritis symptoms, thus improving the arthritis score, and preventing bone destruction and further causing an increase in the population of *L. acidophilus* [45].

In addition to our AA study, the efficacy of LB was evaluated only in a few studies. One study [41] was conducted in a model of induced acute colitis by dextran sulphate sodium (DSS) in male Sprague-Dawley rats. LB was added to the diet of a group with DSS-induced inflammatory processes, and results showed inhibition of the production of IL-6, IL-8, the activity of NF-κB and myeloperoxidase (MPO), and stimulation of IL-13 production. This probiotic strain also reduced the activity of β-glucuronidase (*p* < 0.05), increased counts of *Lactobacilli,* and decreased the number of coliform bacteria, indicating beneficial immunomodulatory and preventive effects in a model of acute colitis [41]. Further, in a rat model of colitis induced by DSS, LB attenuated the DSS-induced signs of an inflammatory process in the colon such as weight loss, diarrhea, infiltration of inflammatory cells associated with decreased colon weight/length ratio, and inhibited gut mucosa destruction and the depletion of goblet cells. Moreover, the strain increased the concentration of anti-inflammatory cytokine IL-10 in mucosal tissue. The protective effects of LB in the DSS-induced colitis model were related to the stimulation of IL-10 and the restoration of goblet cells and indicated it as an excellent candidate to prevent and treat diseases associated with inflammation [39].

Another study showed the preventive administration of LB alone or in combination with prebiotic inulin to rats with N,N-dimethylhydrazine-induced chronic inflammation. The LB administration reduced inflammatory process in the jejunal and colon mucosa, probably indirectly, and involved downregulation of the synthesis of proinflammatory cytokines and suppression of NF-κB activity in mucosal cells [38].

In our experiment with Lewis rats, we evaluated two biometric parameters for describing the development of experimental arthritis and for investigating the treatment effectivity. Both inflammatory and arthritic changes occurring in rats with AA are reflected by hind paw swelling and weight loss in the experimental animals. For both parameters, change of HPV (cHPV) as well as change of body weight (cBW), we have found significant differences between arthritic and healthy animals on day 14, which indicates that the arthritis was already fully developed. After one week (experimental day 21), in the case of both parameters, the differences between healthy and arthritic animals were even more profound, as could be expected in the acute phase of AA. During the chronic phase of arthritis (experimental day 28), we observed regression of the differences between the AA and healthy control animals. According to our findings, the changes in biometric parameters typical for the animal model of AA are also described elsewhere [64,65]. LB administered to arthritic animals improved both parameters. During the experiment, it had a more visible effect for cHPV than cBW. LB administered in combination with MTX helped alleviate the swelling of hind paws and loss of body weight during the whole experiment. The combination therapy was more effective in decreasing inflammation causing hind paw swelling than MTX alone. This finding was proved on days 21 and 28 also by statistical analysis. Similar results were observed for arthritic cachexia suppression but without showing significance. Combinational therapy of *L. casei* with MTX proved decreasing of HPV and improving of the arthritic symptoms similarly [45].

Gamma-glutamyl transferase (GGT) is an enzyme present on the cell surfaces of many bodily tissues and is thought to be one of the pathogenic elements contributing to the inflammatory processes. Increased GGT expression and activity in joint tissue is a reliable experimental indicator of synovial inflammation in experimental arthritis, as in the CIA model. Anti-GGT antibodies are suggested as innovative therapeutic agents for reducing joint deterioration and osteoclast formation in RA patients by neutralizing GGT [66,67]. In addition, Ishizuka, et al. (2007) described the therapeutic effect of neutralizing antibodies against GGT on joint destruction using a CIA in mice [66]. We studied the changes in GGT activity in the spleen and joint tissues in our studies, for example, with bioflavonoid-robinin [68]. As previously shown by Tsiklauri et al. (2021), results from present study also show that the values were elevated differently in the examined tissues (higher values were measured in the spleen compared to joint tissue), and significant differences were shown between arthritic and healthy animals. In this study we also achieved regression of the GGT activity for monotherapy and combination therapy. The highest decrease was seen with combinational therapy in both tissues.

Inflammation causes a release of variable inflammatory mediators and chemokines from damaged blood cells, for example, Il-1β or TNF-α [69]. Inflammatory symptoms, such as edema and hyperalgesia, as well as joint damage and auto-inflammatory illnesses, are mediated mainly by IL-1β [70]. Despite many other studies with probiotics focused on IL-1 β expression during treatment in AA, we have chosen to focus on IL-17A instead, as this cytokine is specific for autoimmune diseases. Like IL-1β, IL-17A is produced by activated adaptive and innate immune cells [71,72], and IL-17 contributes to inflammatory changes seen during RA. Various studies showed the enhancing effect of IL-17 on IL-1β and TNF-α in inducing the production of IL-6 and IL-8, which are proinflammatory cytokines [73,74]. Therefore, we studied IL-17 plasmatic levels in experimental arthritis and its pharmacological modulation with plant compounds such as robinin [68], Fatsiphloginum™ [75], or N-feruloylserotonin [76]. The concentrations of IL-17A in plasma were determined on days 14, 21, and 28. In AA animals, the changes in IL-17A on days 14 and 21 compared to healthy animals are noticeable in all experiments mentioned above. However, on day 28, at the end of the experiment, there were no differences among experimental groups in levels of IL-17A. A significant effect has been shown in reducing the levels of the proinflammatory cytokines (IFN-γ, TNF-α, IL-1β, IL-17, and IL-6) in a group of AA rats treated with *L. casei* [45]. In this experiment, we also observed an effect in reducing levels of IL-17A in plasma by LB at day 21 (not significantly). However, combinational therapy significantly reduced levels of IL-17A during all experimental days. Thus, the addition of LB to MTX caused an enhancing effect on the treatment of AA. Similarly, the combination therapy of MTX with natural compounds proved to be more effective in our previous experiments as well [68,75,76,77].

In the present study with LB, we chose to follow the changes in levels of MCP-1 and MMP-9 caused by arthritis. Joint synovium produces MCP-1 in several inflammatory joint diseases. Synovial release of MCP-1 was implicated in the monocyte recruitment during RA-associated inflammation, with synovium macrophages being the primary source of this cytokine [78]. In RA patients, MCP-1 was indicated as a potent chemotactic agent for monocytes/macrophages and implicated in inducing MMPs secretion [79]. MMPs are enzymes produced in response to pro-inflammatory cytokines such as IL-1 and TNF-α by activated macrophages and fibroblasts. They were shown to be involved in the destruction of articular tissues in RA [80,81]. Similarly, to the findings of the present study, in our previous studies, while evaluating MMP-9 plasmatic levels [77,82], we found its increased levels mainly in the acute phase of AA. In the chronic state of AA, significant differences between healthy and AA animals have also been found [77,82]. To our knowledge, no experimental results have been published evaluating both the MCP-1 and MMP-9 levels in adjuvant arthritis or CIA so far. In the present study, the concentration profiles for MMP-9 and MCP-1 in arthritic animals showed a decreasing trend from day 14 to day 28. As for the positive anti-inflammatory effect of *Lactiplantibacillus plantarum* LS/07 observed in our study, these findings are in good agreement with those reported by Tarapatzi et al. (2022) and Wang et al. (2022) [83,84].

In conclusion, we found the combination treatment to be significantly more effective in improving cHPV compared to MTX alone on days 21 and 28. All three immunological parameters monitored (IL-17A, MMP-9, and MCP-1) did not change by the application of LB in healthy groups of animals. We could thus assume that LB unlikely affected the basal levels of these inflammatory markers. On the other hand, the administration of LB to the AA rats improved their elevated concentration of IL-17A on day 21, although not significantly. MTX was effective in lowering the IL-17A concentrations on days 14 and 21. Finally, the combination of MTX with LB was found to be more effective in treatment of AA rats than that of MTX alone. As for the time course, the increased plasma concentrations of MMP-9 and MCP-1 in AA rats were effectively influenced by the applied combinational therapy at day 14. Overall, the efficacy of the treatment tested under our experimental conditions increased in the following order AA-LB < AA-MTX < AA-LB-MTX. Regarding the efficacy of LB monotherapy, we observed a significant decrease of MMP-9 (day 14) and MCP-1 (day 21) compared to the untreated AA group. The combinational therapy showed an effect in decreasing the activity of GGT in joint tissue. In the spleen tissue, GGT activity was significantly decreased by LB in monotherapy as well as in combination with MTX. Concluding, the findings of the present study indicated a beneficial action of the combinational therapy over the methotrexate therapeutic activity in experimental arthritis with no apparent negative effect of LB in monotherapy on inflammation parameters studied. Particular mechanisms involved in the modulation of immune processes involved in RA pathology need to be further elucidated.

## 4. Materials and Methods

### 4.1. Experimental Animals

Lewis male rats were bred and purchased for this experiment from the Department of Toxicology and Laboratory Animal Breeding, Centre of Experimental Medicine, SAS, Dobrá Voda, Slovak Republic (SK CH 24016) at the age of 5 weeks. The Ethics Committee of the Institute of Experimental Pharmacology and Toxicology, Center of Experimental Medicine SAS in Bratislava, Slovakia (SK UCH 01017) and State Veterinary and Food Administration of Slovak Republic, Bratislava (3144/16-221/3) authorized the protocol for this experiment. Animals were, upon arrival, submitted to quarantine, which lasted 7 days. Housing conditions were standard, 12 h/12 h light/dark regime, humidity 55%, 21–24 °C. Animals had ad libitum access to a standard pallet diet and tap water. The animal housing agrees with EU Convention for the Protection of Vertebrate Animals Used for Experimental and Other Purposes. Animals were sacrificed at the end of the experiment under deep general anesthesia.

### 4.2. Induction of Adjuvant Arthritis

Adjuvant arthritis (AA), based on its characteristics, is a well-established model of inflammation [85]. To induce AA, animals weighting 160–180 g were injected with 0.1 mL suspension of heat-killed *Mycobacterium butyricum* in incomplete Freund’s adjuvant (Difco, Tucker, GA, USA) intradermally at the base of the tail as previously described [68,86].

### 4.3. Bacterial Cultivation

*Lactiplantibacillus plantarum* LS/07 (LB) was grown in MRS broth (de Man, Rogosa and Sharpe broth, Merck, Darmstadt, Germany) at 37 °C. The concentration of viable bacteria was assessed using plate counting on MRS agar (Merck, Darmstadt, Germany). The assay was performed in triplicate. In addition, a stationary overnight culture containing 4 × 10^9^ CFU/mL viable bacteria and bacterial metabolites was used for further experiments. Viable bacteria and bacterial metabolites were applied directly perorally in a volume of 100 μL to experimental animals, and thus each rat received a suspension of LB (0.4 × 10^9^ CFU; see Table 1).

### 4.4. Experimental Design and Treatment

Rats were randomly divided into six experimental groups: 1. a control group of healthy animals (CO); 2. a control group of healthy animals administered with LB daily (100 μL suspension per rat, CO-LB); 3. a group of untreated animals with AA (AA); 4. a group of animals with AA administered with LB daily (100 μL suspension per rat, AA-LB); 5. a group of animals with AA, which was administered MTX twice a week 0.3 mg/kg (AA-MTX); and 6. a group of animals with AA, which received a combination of MTX and LB in the same doses and regimen as in monotherapies (AA-LB-MTX) (Table 1). In our experiment, we used the following number of rats in each group: CO: *n* = 10, AA: *n* = 9, CO-LB: *n* = 11, AA-LB: *n* = 11, AA-MTX: *n* = 11, AA-LB-MTX: *n* = 10. The probiotic strain and the drug were administered perorally during the experiment (28 days). On days 14 and 21, blood samples were taken from the retro-orbital plexus under light Zoletil^®^/Xylazine anesthesia. On the 28th day, animals were sacrificed under deep Zoletil^®^/Xylazine anesthesia and blood for plasma preparations was withdrawn. In addition, tissues such as spleen and joints were collected from each rat for further analysis. All samples were stored at −70 °C until analysis.

**Table 1:** Experimental design of animal groups with their treatment.

Group	Treatment
**CO**	vehiculum, daily
**CO-LB**	100 μL suspension of LB, daily
**AA**	vehiculum, daily
**AA-LB**	100 μL suspension of LB, daily
**AA-MTX**	MTX 0.3 mg/kg, twice a week, vehiculum, daily
**AA-LB-MTX**	MTX 0.3 mg/kg, twice a week + 100 μL suspension of LB, daily

CO—control group, AA—adjuvant arthritis, LB—*Lactiplantibacillus plantarum* LS/07, MTX—methotrexate, vehiculum—MRS broth without LB.

### 4.5. Evaluation of Experimental Arthritis

Biometrical parameters such as volume of hind paws and body weight were measured to evaluate changes during the progression of the disease on the 14th, 21st, and 28th day after initial immunization with *M. butyricum*. Change of hind paw volume (cHPV) was expressed as the average of the elevation of percentage (%) of the hind paw volume of each rat, compared with HPV measured at day 1 using a water plethysmometer (UGO BASILE, Comerio-Varese, Italy). The measured HPV on the selected day was divided by the HPV on day 1 and expressed in percentage according to the following formula:

([n Day]/[Day 1]) × 100 − 100 = value [%](1)

The change of body weight (cBW) of the animals was assessed daily. The changes in body weight are shown as the average of weight gain [g]. Weight measured on the day (n—day 14, 21, and 28) minus weight measured on day 1. The mathematical formula we used, as described before [68], is as follows:

[n Day] − [Day 1] = value [g](2)

### 4.6. Biochemical Evaluation of Markers of Inflammation in Plasma

All markers of inflammation in plasma were measured by using an enzyme-linked immunosorbent assay (ELISA). For plasmatic concentrations analysis of monocyte chemotactic protein-1 (MCP-1) and interleukin-17A (IL-17A), we used kits by eBioscience^®^ (Waltham, MA, USA), and for metalloproteinase 9 (MMP-9) we applied kit by R&D Systems (Minneapolis, MN, USA). The assay procedures were applied as described in the product manuals.

### 4.7. The Determination of Gamma-glutamyl Transferase Activity in the Hind Paw Joint and Spleen Tissue

To measure the activity of γ-glutamyl transferase (GGT) on day 28 in the spleen and hind paw joint tissue homogenates, the method of Orlowski and Meister (1970) [87] and modified by Ondrejickova et al. (1993) [88] was used as in our previous protocol [68]. The tissues were homogenized in a buffer (2.6 mM of NaH_2_PO_4_, 50 mM of Na_2_HPO_4_, 68 mM of NaCl, 15 mM of EDTA; pH 8.1) at 1:9 (*w*/*v*) by Ultra Turax TP 18/10 (Janke and Kunkel) for 1 min at 0 °C. The biochemical substrates (44 mM of methionine and 8.7 mM of L-γ-glutamyl-p-nitroanilide) were then dissolved in isopropyl alcohol (65%) to final concentrations of 2.5 mM and 12.6 mM, respectively. The samples were incubated for one hour at 37 °C, and then we added 2.3 mL of cold methanol to stop the reaction. Tubes were centrifuged at 5000 rpm for 20 min (Centrifuge Eppendorf). The supernatant’s absorbance (product p-nitroaniline) was measured on a spectrophotometer Specord 40 (Analytikjena, Jena, Germany) at 406 nm. Solution mixes without or without substrate or acceptor were used as blanks. The activity was calculated based on absorbance measurement using a calibration coefficient.

### 4.8. Statistical Analyses

Mean and SEM values were calculated for each parameter in each group (from 6 to 11 animals in each experimental group). Statistically significant differences among treated, untreated, and control groups were tested using parametric analysis of variance (ANOVA). Post-hoc tests (Tukey-Kramer) were applied in situations where differences among groups were significant at the level of significance α = 0.05. After post-hoc testing, the following significance levels were specified: extremely significant (*p* < 0.001; ***/+++/###, very significant (*p* < 0.01; **/++/##), significant (*p* < 0.05; */+/#), and not significant (*p* > 0.05). The untreated healthy controls (CO) were compared to treated healthy controls (CO-LB). The untreated arthritis group (AA) was compared with healthy control animals (CO) (*) and with the treated arthritis groups (AA-LB, AA-MTX, and AA-LB-MTX) (+)). The Group of arthritic animals treated with MTX (AA-MTX) was compared to the treated arthritis group also receiving *L. plantarum* (AA-LB-MTX) (#).

## Data Availability

The experimental data are reported here: https://figshare.com/articles/dataset/Pruzinska_et_al_Lactobacillus_plantarum_LS07_MTX_xlsx/20715553 (accessed on 13 December 2022).

## Sample Availability

Samples of the compounds are available from the authors.
